# Supplementary material for: Construction of Genetic Linkage Map and Mapping QTL Specific to Leaf Anthocyanin Colouration in Mapping Population ‘Allahabad Safeda’ × ‘Purple Guava (Local)’ of Guava (Psidium guajava L.)
Source: Plants (Basel). 2022 Aug 2;11(15):2014. doi: 10.3390/plants11152014 (PMC9370526; doi:10.3390/plants11152014)
Supplement: Supplementary file 1 [file plants-11-02014-s001.zip › plants-1840791-supplementary.pdf]

plants

MDPI

Article

Construction of Genetic Linkage Map and Mapping QTL Specific to Leaf Anthocyanin Colouration in Mapping Population 'Allahabad Safeda' × 'Purple Guava (Local)' of Guava (*Psidium guajava* L.)

Harjot Singh Sohi [1,*], MIS Gill [1], Parveen Chhuneja [2], N.K. Arora [1], Sukhjinder Singh Maan [1] and Jagmohan Singh [3]

Supplementary Materials

Table S1. Anthocyanin Reflective Index (*ARI*1) of the guava parents and their F1 hybrids at LSD (p≤0.05).

Sr. No.	Varieties/F1 hybrids of Guava	Anthocyanin Reflective index value (*ARI*1)
1	Allahabad Safeda 'AS'	0.053
2	Purple Guava (local) 'PG'	0.266
3	Seedless 'SL'	0.013
4	CISH G-1	0.172
5	'AS' x 'PG'	0.214
6	'AS' x 'CISH G-1'	0.125
7	'AS' x 'SL'	0.027
	**LSD (p≤0.05)=0.02**	

Table S2. A candidate gene markers *MdMYB*.

Sr No	Locus	Repeat motif	Primer sequence (5′ to 3′)	Tm	Reference allelic Size (bp)	Amplification
1	MdMYB9	(CT)15	F: TCCATAACCTTCTTGGTAACA<br>R: AAGGAGAATCGGCTTTCGAT	52.1<br>54.0	606	NA
2	MdMYB10	-	F: CGAGTGCTAATAGCAACC<br>R: CCTCTGTTGGCCGAATACAC	50.4<br>55.8	1945	NA
3	MdMYB10 (F1, R1)	-	F: AATGGCCTACCACATCATTG<br>R: AATGGCCTACCACATCATTG	52.9<br>52.9	786	P
4	MdMYB10 (F2, R2)	-	F: AGGTGCCTGGACTCGAGAGGA<br>R: CGACGACGTTTGTGGTGGCG	62.0<br>61.7	1182	NA
5	MdMYB17	(T)14/(AT)10	F: TGCTCCTCTCTAGCTATTGCA<br>R: AAGACTCACAAACTAGCTGTC	55.5<br>54.7	205	NA

Five anthocyanin apple candidate gene markers referred by Chagne *et al.* (2007) were tested on parent AS, PG and F1 hybrids (H) of cross combination (I). # NA- Non-amplified, P- Polymorphic.

Table S3. List of SSR primer pairs used for the construction of linkage map in guava.

Sr. No.	Locus	Repeat motif	Primer sequence (5′ to 3′)	Tm	Reference allelic Size (bp)
1	mPgCIR1	$(GA)_{17}$	F: TAGTGCTTTGGTTGCTT<br>R: GCAGGTGGATATAAGGTC	48.8<br>49.3	237
2	mPgCIR2	$(GA)_{20}$	F: AGTGAACGACTGAAGACC<br>R: ATTACACATTCAGCCACTT	51.5<br>49.2	224
3	mPgCIR3	$(GA)_{40}$	F: TTGTGGCTTGATTTCC<br>R: TCGTTTAGAGGACATTTCT	46.6<br>47.9	158
4	mPgCIR4	$(GA)_{25}$	F: TTCAGGGTCTATGGCTAC<br>R: CAACAAGATACAGCGAACT	50.6<br>50.0	148
5	mPgCIR5	$GA)_{31}$	F: GCCTTTGAACCACATC<br>R: TCAATACGAGAGGCAATA	47.8<br>47.3	252
6	mPgCIR7	$(CA)_{13}AA(GAA)_3$	F: ATGGAGGTAGGTTGATG<br>R: CGTAGTAATCGAAGAAATG	47.8<br>45.4	149
7	mPgCIR8	$(GA)_{12}$	F: ACTTTCGGTCTCAACAAG<br>R: AGGCTTCCTACAAAAGTG	49.5<br>49.3	214
8	mPgCIR9	$(GA)_{19}$	F: GCGTGTCGTATTGTTTC<br>R: ATTTTCTTCTGCCTTGTC	48.6<br>47.4	173
9	mPgCIR10	$(CT)_{12}$	F: GTTGGCTCTTATTTTGGT<br>R: GCCCCATATCTAGGAAG	47.4<br>48.4	261
10	mPgCIR11	$(CT)_{17}$	F: TGAAAGACAACAAACGAG<br>R: TTACACCCACCTAAATAAGA	47.5<br>47.9	298
11	mPgCIR13	$AC)_{12}(AT)_4G(GA)_2$	F: CCTTTTTCCCGACCATTACA<br>R: TCGCACTGAGATTTTGTGCT	52.7<br>54.9	245
12	mPgCIR14	$(GA)_{11}$	F: TAAACACAACAAGGGTCA<br>R: CAGTTTTCATATCGTCCTC	48.5<br>49.2	185
13	mPgCIR15	$(GA)_8GG(GA)_9$	F: CCGATCATCTCTTTCTTT<br>R: TCTAATCCCCTGAGTTTC	47.9<br>46.3	147
14	mPgCIR16	$(TC)_{25}$	F: AATACCAGCAACACCAA<br>R: CATCCGTCTCTAAACCTC	48.5<br>49.2	292
15	mPgCIR17	$(CT)_{23}$	F: CCTTTCGTCATATTCACTT<br>R: CATTGGATGGTTGACAT	47.1<br>46.7	231
16	mPgCIR18	$(GA)_{23}$	F: TAAGCTGCATGTGTGC<br>R: ATGGCTTTGGATGAAA	50.0<br>45.2	195
17	mPgCIR19	$(CT)_{16}$	F: AAAATCCTGAAGACGAAC<br>R: TATCAGAGGCTTGCATTA	47.1<br>47.6	274
18	mPgCIR20	$(CT)_{14}(CA)_{17}$	F: TATACCACACGCTGAAAC<br>R: TTCCCCATAAACATCTCT	49.4<br>47.2	266
19	mPgCIR21	$(AG)_{15}GG(AG)_7$	F: TGCCCTTCTAAGTATAACAG<br>R: AGCTACAAACCTTCCTAAA	48.8<br>48.2	154
20	mPgCIR22	$(GT)_9(GA)_{14}$	F: CATAAGGACATTTGAGGAA<br>R: AATAAGAAAGCGAGCAGA	46.8<br>48.5	235
21	mPgCIR23	(TA)4$(GT)_7$	F: GTCTATACCTAATGCTCTGG<br>R: CCCAGGAAAATCTATCAC	49.2<br>47.0	185
22	mPgCIR25	$(GA)_{24}$	F: GACAATCCAATCTCACTTT<br>R: TGTGTCAAGCATACCTTC	47.3<br>49.4	124
23	mPgCIR26	$(GT)_2(GA)_{17}$	F: CTACCAAGGAGATAGCAAG<br>R: GAAATGGAGACTTTGGAG	49.3<br>47.5	185
24	mPgCIR30	$(GA)_{30}$	F: CTCAAAGCACTATCATGTCG<br>R: CCTTGTGGGTTCTCTTTTG	51.0<br>51.1	176

Sr. No.	Locus	Repeat motif	Primer sequence (5′ to 3′)	Tm	Reference allelic Size (bp)
25	mPgCIR31	$(GA)_{24}$	F: TCTCACTGATGCAACTTTTC<br>R: CCCATTTTCATCTCAAAGTC	50.6<br>49.0	128
26	mPgCIR32	$(GA)_{16}$	F: CGCCTTTCGTAAAAGAAGT<br>R: TCATATACTCGGACAAAACG	50.5<br>49.6	100
27	mPgCIR39	$(GA)_{17}$	F: GCTCACCTTACTCATTCAGC<br>R: CTGTTGCTAAGAGCTTTCGT	53.3<br>52.8	155
28	mPgCIR40	$(GTA)_{6}$	F: TGAATCTCCAGTGTCTTATCG<br>R: TGATTTCAACTGCGTATGTC	51.6<br>50.6	141
29	mPgCIR41	$(GA)_{13}$	F: AAGTGGTGTCAGCAACTACC<br>R: CTTAGTTTGACCGCTCCAGT	54.9<br>54.6	136
30	mPgCIR42	$(GA)_{9}$	F: CTCACCCAAAATCTACACAAG<br>R: AAGGGACTGGACGATGTT	51.2<br>53.4	107
31	mPgCIR46	$(GA)_{36}$	F: ATAGAACGCCATGTTACCAA<br>R: CAGGCTTATCTGTTACACCA	51.6<br>51.9	159
32	mPgCIR89	$(GA)_{16}$	F: TCGTCCAGAATCTTAAAAGC<br>R: AACTAGCATGTGACCAGGAG	50.1<br>54.2	274
33	mPgCIR91	$(GA)_{16}$ / $(GGGA)_{3}$	F: GCGGTGGATTTGAATTTAG<br>R: CCAAGTAACCCACAACAATA	49.2<br>50.1	125
34	mPgCIR93	$(GA)_{16}$	F: GCATCATGTGTTTGAACGAT<br>R: AAGTGTGCGTTCTCCATCT	51.5<br>54.0	123
35	mPgCIR94	$(GA)_{18}$/$(GT)_{6}$	F: CAACCTTCCCGTGATTATT<br>R: CTAGCTTCTTCAGTGGGAAC	49.8<br>52.7	254
36	mPgCIR98	$(GA)_{15}$	F: CATCAACTTTCCAGGCATA<br>R: CCATTCAGTCGGTTTGAC	49.9<br>50.9	127
37	mPgCIR99	$(GA)_{20}(GT)_{14}$	F: TCAAAGTCCAAAACTCATGC<br>R: GGGATGGAGTAAAGATGAAA	51.1<br>49.4	220
38	mPgCIR105	$(GA)_{30}$ / $(GAAAGA)_{3}$	F: CCTCCTTCGCTCTACATAAA<br>R: ATTACCCCACGAACATATCA	51.4<br>50.9	235
39	mPgCIR110	$(GT)_{33}$	F: GCCCCATTCTAAGAGACAAT<br>R: GAATGAAACCAGGTGTAGCA	52.0<br>52.7	118
40	mPgCIR111	$(GA)_{19}$	F: CAACCTCGTTTGAGTCTTCT<br>R: AACATCATTGGGACCATTC	52.2<br>50.4	115
41	mPgCIR137	$(GA)_{18}$	F: GGGGAATGCAGAGATTGT<br>R: AGATGATGGTCTCGCTTTT	52.2<br>51.4	100
42	mPgCIR139	$(GT)_{9}$ / $(GA)_{9}$	F: ATAATCCCCTCCCATAACTA<br>R: CCAACTCAACATGAGAAGC	49.3<br>51.2	207
43	mPgCIR140	-	F: GTGGTGAAGGAGTAAAGCTG<br>R: GCAGTATAAAGCAACAGATGG	53.2<br>51.6	-
44	mPgCIR146	$(GT)_{11}$	F: CGGATATTAGCAGGAGAAAA<br>R: AATGTTTGACGACTCGAAAG	49.4<br>50.8	110
45	mPgCIR150	$(GA)_{15}$	F: CCTAGTGACTCGAAGCAATC<br>R: TTGAGCCCTAGCATAGACAG	52.7<br>53.8	108
46	mPgCIR153	$(GA)_{20}$	F: GCCTCTGGTAAATCTGTTGA<br>R: ACATACGGATCAAGTCCAAA	52.2<br>51.3	115
47	mPgCIR157	$(GA)_{21}$ / $(GGAA)_{3}$	F: AACCACCAAACCATACACC<br>R: CGACCAACCCTACATTCTG	52.8<br>52.8	209
48	mPgCIR160	-	F: TGGCTATAAGAATGGGAGAT<br>R: GACGAGCTTAGCCTCTGAAT	50.2<br>52.4	-
49	mPgCIR161	-	F: TCTCAAGGACCAACAAGAAG<br>R: AGGACTTAGCTTGGGTTTTC	52.0<br>52.4	-

Sr. No.	Locus	Repeat motif	Primer sequence (5′ to 3′)	Tm	Reference allelic Size (bp)
50	mPgCIR165	$(GA)_{11}$	F: TAAGGGATTCATTTCCGAGT<br>R: CTGGTGTGACGATGACTTTT	50.8<br>52.8	124
51	mPgCIR175	$(GA)_{16}$	F: GCATTATGTGCCAAGCAA<br>R: TGCCAAGGTGTAATGTTGTA	51.3<br>51.8	104
52	mPgCIR176	$(GA)_{15}$ / $(GA)_{10}$ / $(GT)_{18}$ / $(CGTC)_{3}$	F: TTCTCAATGATAGGTTACGG<br>R: ATGACTATTCTCCACCAGAT	49.1<br>50.1	151
53	mPgCIR179	$(GA)_{16}$	F: GGGTCTCGACTAAAGAAGGA<br>R: CCTCCATTTGCATCAACTTT	53.3<br>51.3	147
54	mPgCIR183	$(GA)_{27}$	F: GAAGAACAGAGACGCACCT<br>R: GCGCTAGGCTATAAATCATC	54.5<br>50.7	183
55	mPgCIR186	$(GA)_{18}$ / $(GA)_{8}$ / $(GA)_{15}$	F: TCGAGCGGTTCTTGTCCT<br>R: ATCTTCTCCTCCTTCCATCC	56.1<br>53.3	224
56	mPgCIR188	$(GA)_{9}$	F: TGGATGAATCAGGAGGATTA<br>R: TTGTGGGGAAGAAACTACTG	50.1<br>52.1	189
57	mPgCIR194	$(GA)_{20}$	F: GCAGAGAATCGAAGCACTA<br>R: GCAAGCACAGGTTCTACTTT	51.9<br>53.1	172
58	mPgCIR196	$(GA)_{17}$	F: TAACACCACGTCCATCAATA<br>R: GCGTTAAGCTACTTTGGCTA	51.0<br>52.7	120
59	mPgCIR198	$(GA)_{22}$ / $(GGTTGA)_{3}$	F: CTCGATCAGAAGAACAACATC<br>R: ACTGTTCCTGATGGCTCTC	50.9<br>54.1	176
60	mPgCIR201	$(GA)_{19}$	F: TTTGCCTTCGAGCTTCTACT<br>R: ACAATTTCGTGGGCTCGT	53.8<br>54.8	133
61	mPgCIR208	$(GA)_{29}$ / $(GAG)_{5}$	F: AGCACGATGTAGGAAGAGAC<br>R: CGGCAAGCTCAACAATATAA	53.9<br>50.7	242
62	mPgCIR209	-	F: CTAAAGCCACATCCAGCA<br>R: CTAACATTTGCCTTCTACAGC	52.4<br>51.5	-
63	mPgCIR216	-	F: GATGGAACTGCAATGTATGA<br>R: AGACCTGCTCTGACTTGTGA	50.7<br>55.6	-
64	mPgCIR227	$(GA)_{16}$	F: GGGATGCTCAAAACTGTAAG<br>R: CCTGTTACATTGACGAATCA	51.2<br>50.1	168
65	mPgCIR228	$(GT)_{14}$/$(GA)_{18}$	F: CAGAACAAAGAAGAGGATCTG<br>R: TGGATCAGTAGAATCGTTTG	50.7<br>49.6	153
66	mPgCIR229	$(GA)_{26}$	F: GTCAAGCATACCTTCACCTC<br>R: CATCTCATCAACAGATTGGA	53.0<br>49.6	143
67	mPgCIR233	-	F: GACTGAAGACCCAAATACCA<br>R: TTAGGCTGAAATGCTCCTTA	51.9<br>51.1	-
68	mPgCIR234	$(GT)_{11}$	F: TTGGCTTGTCAACTACTGG<br>R: GCATCTCTTTCATTGGTGAT	52.4<br>50.5	159
69	mPgCIR235	$(GA)_{28}$ / $(GAGT)_{4}$	F: TCTTCTGGACAACAAATTCC<br>R: GATTTAAGGCAAAGGATCG	50.3<br>48.7	204
70	mPgCIR236	$(GA)_{23}$	F: ACTCATATTCCGTTTGCATC<br>R: GAATTAACGACGAGTTCCAC	50.5<br>51.1	164
71	mPgCIR237	$(GA)_{16}$	F: AGATTCCATCTGCGATTGT<br>R: GCGGATCAAAACCTAATCT	51.4<br>49.9	101
72	mPgCIR242	$(GA)_{12}$	F: TTAAGGTGGGACCAAGAAG<br>R: GACGTATCGGATCAAGTTTC	51.6<br>50.7	178
73	mPgCIR243	$(GA)_{29}$	F: ACAGCAGGACACAAAGGA<br>R: GCTCTGAGGTGGTTTTCAT	53.9<br>52.5	174
74	mPgCIR246	$(GA)_{21}$	F: GAATTACAAATGCCTTGTCC<br>R: GCTCTAAAGTGCAACCAAAG	49.5<br>51.9	132

Sr. No.	Locus	Repeat motif	Primer sequence (5′ to 3′)	Tm	Reference allelic Size (bp)
75	mPgCIR247	$(GA)_{20}$	F: CTTTGCACTCAATGTGTTTG<br>R: CCTTGTCACTCTTATAGCATCA	50.4<br>51.9	191
76	mPgCIR249	$(GA)_{21}/(GAA)_{5}$	F: TTTGTCTGGTCGTCCTAGTT<br>R: CTTCAGTCCATCAGCAAAAT	53.2<br>50.7	259
77	mPgCIR251	$(GA)_{21}$	F: TTTACAGCTTCTTCCTTTGC<br>R: GTCGTCTACTTCGATGGTTC	50.9<br>52.6	196
78	mPgCIR252	-	F: AGGAAGAAAGGGTGGTCTAT<br>R: CTCCTGTTCTCGTTCTTAGG	52.7<br>52.1	-
79	mPgCIR253	$(GA)_{29}$	F: TGCCCTTAGCCTATAACTCA<br>R: CTTGTGGTCCAAGATGCTAT	52.7<br>52.2	197
80	mPgCIR255	$(GA)_{27}/(GA)_{23}$	F: TTGTTCTCTTTGTGGTTCCT<br>R: GTGACAACGTCAGTGGAGA	51.7<br>54.4	210
81	mPgCIR256	$(GA)_{12}$	F: AGGTGCATGATTACGATTCT<br>R: CGAGGTTCTTGATGTTGTCT	51.4<br>52.3	212
82	mPgCIR257	$(GA)_{29}$	F: CGACTCATTTTCTGGTCTGT<br>R: CAACCACCTTCATCAATTTC	52.3<br>49.5	203
83	mPgCIR265	$(TC)_{24}$	F: CTACAGCGAATTTCTCGATT<br>R: GTCAACGGATCAATGTGG	50.2<br>51.0	144
84	mPgCIR284	$(AC)_{10}$	F: AACCCTTTCGGGGTCAAG<br>R: GATCCGATTGCGGAAGAG	54.9<br>53.1	90
85	mPgCIR285	-	F: CTTTGGGTGTCTCTGTGTCT<br>R: CAGCCAGGGCATAAGTATAG	54.3<br>52.4	-
86	mPgCIR287	$(AG)_{35}/(TA)_{6}$	F: GCTGGTGCAAAAGTAGTCA<br>R: GCAGTTCTTTTCCTTCTAACC	53.0<br>51.4	266
87	mPgCIR290	$(GA)_{23}$	F: AAATAGGAAGTAACCTGTGC<br>R: CCTGAACTTCTGTGTTTATATT	49.9<br>48.2	185
88	mPgCIR298	-	F: GCGTATGTCATTCCATGTG<br>R: CAGGAATATGATGCTGGAAG	51.3<br>50.6	-
89	mPgCIR321	$(CT)_{19}$	F: TTTTGGCCTGGGAATATAG<br>R: TAAAACGAAAGCAGAAAACC	49.4<br>49.1	129
90	mPgCIR334	$(AG)_{26}$	F: GGATCTAACCGACCTTTCTT<br>R: AAACAGGACTGAGTTTCGAG	51.6<br>52.2	190
91	mPgCIR343	$(AG)_{20}$	F: CACTCACAGCATCGTAAA<br>R: GAAGTGGCAAGAATATCTGT	49.6<br>49.9	250
92	mPgCIR347	-	F: CTCTGAAAGGGAGAGGACTT<br>R: AGAATCTTCGCCTATTGCTT	53.4<br>51.7	-
93	mPgCIR352	$(GA)_{25}$	F: TCGTAGTTAAACGAGTGATGC<br>R: GGACCAAACAGATATTCCAA	52.5<br>49.9	181
94	mPgCIR361	-	F: CTCATGTCCCTTATCAATGT<br>R: AATGTCTCGGTCAATTAGGA	51.9<br>50.8	-
95	mPgCIR377	-	F: CCAAACAAGGATTCACAAAT<br>R: TGTGGTTTAGTTCTTAGTTTTGC	48.9<br>51.6	-
96	mPgCIR378	$(CT)_{10}/(CT)_{6}$	F: GTCACTTGCCTGTTGATTTT<br>R: GCTTTCTCATTTCTTAACCAAG	49.9<br>51.4	162
97	mPgCIR392	$(AG)_{10}$	F: AACTGTTTCTCTGGTTTGATG<br>R: ACGAAACGTCCTTGTTCTTA	51.6<br>50.9	107
98	mPgCIR404	$(GA)_{32}$	F: CTGCAACACATTGAACATTT<br>R: ATTCAGGCTTCTGGTTTGT	49.7<br>51.9	210
99	mPgCIR405	-	F: GAGATTTGGTTGAGGAAGGT<br>R: TTTCAAAATCAAGAGCCAAC	52.2<br>49.1	-

Sr. No.	Locus	Repeat motif	Primer sequence (5′ to 3′)	Tm	Reference allelic Size (bp)
100	mPgCIR414	$(AG)_6/(GA)_{17}$	F: AACAACACGCTTTGAAGTTT<br>R: CCCAGAAAGATGAGACAAAG	51.2<br>50.7	230
101	mPgCIR418	$(TC)_{24}/(TCT)_6$	F: ACGTGACCTTCTCCAACAT<br>R: ACTGGATCGACTGAAGATGA	53.7<br>52.8	228
102	mPgCIR422	$(GA)_{10}$	F: CCGAAAACACAAGGGTTC<br>R: AGGTTCAAATGGTTTGTGG	51.4<br>50.9	236
103	mPgCIR437	$(AG)_{10}$	F: ACAACAGTTCTGATCCCAAA<br>R: CTCGGAGACACAGAGGTCTA	51.8<br>55.1	153
104	mPgCIR441	$(AC)_{10}$	F: TAGGTATGGTTTGAAAGCTC<br>R: GTCTTTCTGCAAATATCCAT	49.6<br>47.9	179
105	mPgCIR446	-	F: GTGACCACTTTCTCAAAGCTA<br>R: ATCTCCATTTCTTGCTCGT	52.5<br>51.4	-
106	mPgCIR448	$(GT)_6$	F: GGAGAGTAGGGTTTCAAAGG<br>R: CGTTTGTTGGACTTGGAG	52.4<br>51.1	114

For the present study, 106 SSR primer pairs (Table 1) belonging to same series were selected from Genome Database for guava (http://neiker.net/neiker/guavamap) and the published literature. The SSR primers were synthesized through Integrated DNA technologies (IDT). The 106 SSR primers were used for generating a linkage map.

Table S4. Anthocyanin Reflective Index (*ARI*1) values of F1 hybrids (‘AS’ x ‘PG’).

F1 Hybrids 'AS' x 'PG'	Anthocyanin Reflective Index (ARI1)
1	0.238
2	0.191
3	0.225
4	0.267
5	0.273
6	0.284
7	0.175
8	0.226
9	0.177
10	0.177
11	0.238
12	0.228
13	0.191
14	0.224
15	0.185
16	0.165
17	0.227
18	0.239
19	0.231
20	0.190
21	0.165
22	0.212
23	0.215
24	0.191
25	0.185
26	0.169
27	0.192
28	0.184
29	0.255

30	0.192
31	0.191
32	0.187
33	0.245
34	0.196
35	0.238
36	0.195
37	0.272
38	0.251
39	0.218
40	0.184
41	0.228
42	0.161
43	0.215
44	0.251
45	0.226
46	0.161
47	0.187
48	0.239
49	0.247
50	0.248
51	0.227
52	0.267
53	0.227
54	0.229
55	0.185
56	0.232
57	0.244
58	0.210
59	0.179
60	0.200
61	0.165
62	0.228
63	0.211
64	0.240
65	0.213
66	0.247
67	0.224
68	0.272
69	0.218
70	0.201
71	0.247
72	0.234
73	0.247
74	0.225
75	0.197
76	0.187
77	0.247
78	0.189
79	0.241
80	0.228

81	0.238
82	0.244
83	0.212
84	0.213
85	0.242
86	0.241
87	0.226
88	0.211
89	0.194
90	0.211
91	0.211
92	0.190
93	0.211
94	0.218
95	0.214
96	0.211
97	0.211
98	0.214
99	0.203
100	0.218
101	0.210
102	0.211
103	0.228
104	0.218
105	0.196
106	0.218
107	0.205
108	0.204
109	0.239
110	0.271
111	0.205
112	0.237
113	0.232
114	0.261
115	0.232
116	0.175
117	0.252
118	0.257
119	0.230
120	0.256
121	0.254
122	0.206
123	0.210
124	0.233
125	0.205
126	0.193
127	0.176
128	0.211
129	0.176
130	0.212
131	0.205

132	0.205
133	0.173
134	0.212
135	0.176
136	0.211
137	0.170
138	0.211
139	0.177
140	0.210
141	0.190
142	0.209
143	0.174
144	0.218
145	0.205
146	0.189
147	0.191
148	0.185
149	0.251
150	0.244
151	0.192
152	0.187
153	0.272
154	0.198
155	0.231
156	0.253
157	0.214
158	0.215
159	0.187
160	0.205
161	0.222
162	0.275
163	0.205
164	0.252
165	0.174
166	0.198
167	0.218
168	0.218
169	0.213
170	0.212
171	0.170
172	0.204
173	0.204
174	0.218
175	0.212
176	0.252
177	0.175
178	0.205
179	0.213
180	0.219
181	0.211
182	0.257

183	0.205
184	0.214
185	0.232
186	0.210
187	0.218
188	0.210
189	0.178
190	0.211
191	0.218
192	0.212
193	0.213
194	0.204
195	0.204
196	0.170
197	0.219
198	0.247
199	0.238
200	0.205
201	0.211
202	0.210
203	0.232
204	0.216
205	0.204
206	0.239
207	0.238
208	0.172
209	0.173
210	0.218
211	0.210
212	0.257
213	0.211
214	0.205
215	0.205
216	0.241
217	0.214
218	0.240
219	0.218
220	0.205
221	0.228
222	0.210
223	0.239
224	0.198
225	0.196
226	0.210
227	0.236
228	0.218
229	0.196
230	0.176
231	0.212
232	0.219
233	0.172

234	0.205
235	0.211
236	0.176
237	0.205
238	0.214
**Mean**	**0.214**
**Standard Deviation**	**0.025562314**
**Skewness**	**0.203954091**
**Kurtosis**	**-0.280135581**

Table S5. Composition of CTAB Extraction Buffer.

Components	Working Concentration
CTAB	1.5%
Tris HCl (pH 8.0) `	100 mM
NaCl	1.4 M
EDTA (pH 8.0)	20 mM
β-Mercaptoethanol	2%
Polyvinylpyrolidone (PVP)	2%

The final pH of the buffer was adjusted to 8.0.

Table S6. Temperature profile used in PCR.

Step	Temperature (°C)	Time (minutes)	No. of cycles
Initial denaturation	94	4	1
Denaturation	94	1	
Annealing	50-60	1	35
Elongation	72	1	
Final Extension	72	7	1
Pause	4	-	-

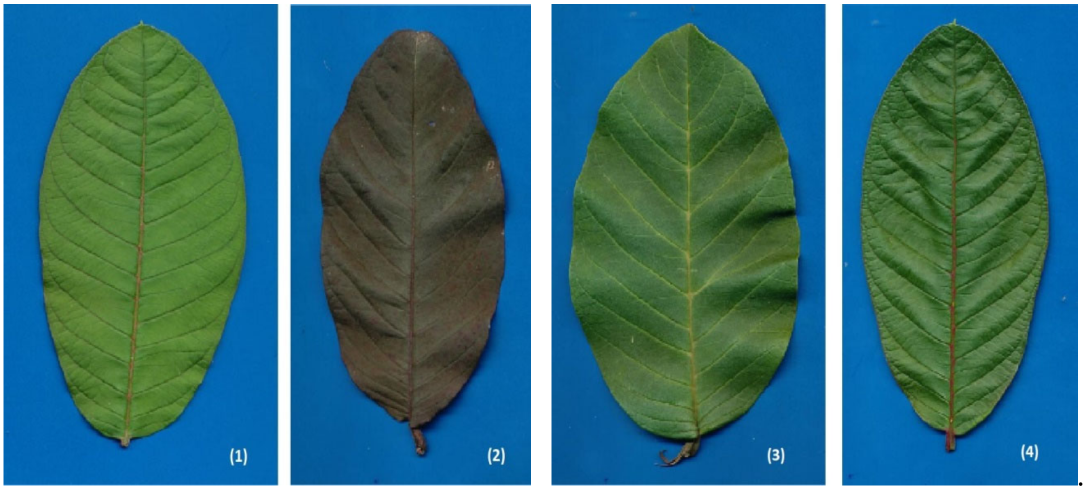

Figure S1. Variation in leaf colour of different guava parents used for obtaining F1 population viz. (1) 'AS' (2) 'PG' (3) 'SL' (4) 'CISH G-1'.

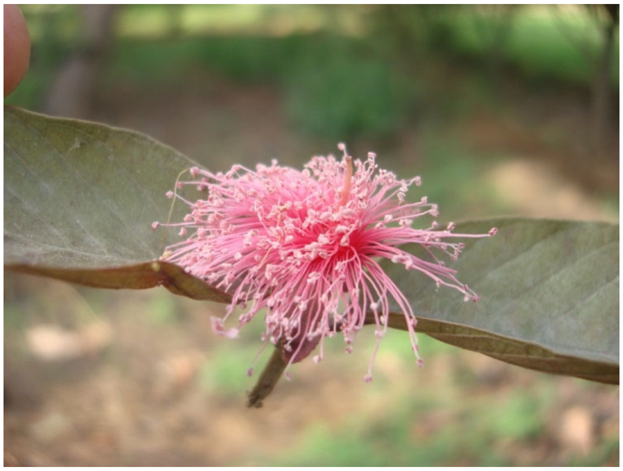

Figure S2. Selection of pollen flower {(Purple guava (local) 'PG'} for the pollination.

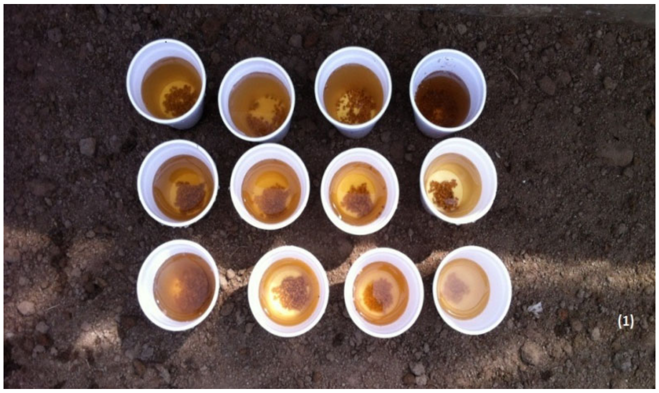

Figure S3. Extraction and Soaking of F1 hybrids (H) seeds obtained from cross combination (I) 'AS' x 'PG'.

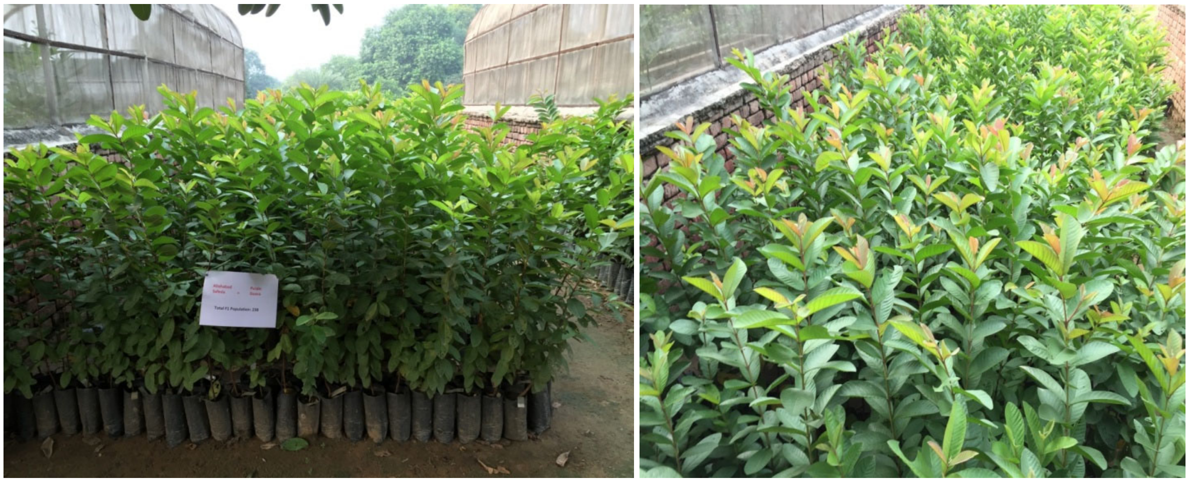

Figure S4. Seggregating 238 hybrids F1 hybrids (H) used as mapping population maintained in old orchard of Punjab Agricultural University,141004, India.

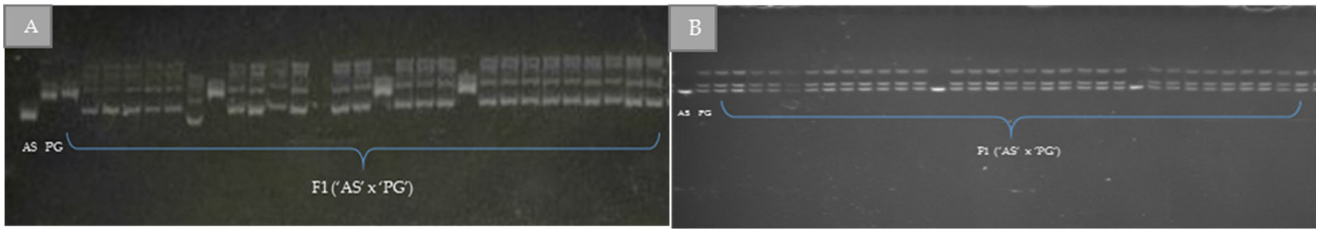

Figure S5. PCR amplification profile of (A) mPgCIR208 and (B) MdMYB10F1 on two parents [AS= Allahabad Safeda, PG= Purple guava (Local)] and their segregating F1 progenies.
